# Studying microstructure and microstructural changes in plant tissues by advanced diffusion magnetic resonance imaging techniques

**DOI:** 10.1093/jxb/erx106

**Published:** 2017-04-08

**Authors:** Darya Morozov, Iris Tal, Odelia Pisanty, Eilon Shani, Yoram Cohen

**Affiliations:** 1 School of Chemistry, The Sackler Faculty of Exact Sciences, and; 2 Department of Molecular Biology and Ecology of Plants, Tel Aviv University, Ramat Aviv, Tel Aviv 66978, Israel

**Keywords:** Adventitious roots, auxin, diffusion MRI (DWI), diffusion tensor imaging (DTI), double-pulsed-field-gradient (d-PGSE) MRI, hypocotyl structure, magnetic resonance imaging (MRI), microstructure, plant development, *q*-space diffusion MRI (QSI), tomato, *Solanum lycopersicum*.

## Abstract

As sessile organisms, plants must respond to the environment by adjusting their growth and development. Most of the plant body is formed post-embryonically by continuous activity of apical and lateral meristems. The development of lateral adventitious roots is a complex process, and therefore the development of methods that can visualize, non-invasively, the plant microstructure and organ initiation that occur during growth and development is of paramount importance. In this study, relaxation-based and advanced diffusion magnetic resonance imaging (MRI) methods including diffusion tensor (DTI), *q*-space diffusion imaging (QSI), and double-pulsed-field-gradient (d-PFG) MRI, at 14.1 T, were used to characterize the hypocotyl microstructure and the microstructural changes that occurred during the development of lateral adventitious roots in tomato. Better contrast was observed in relaxation-based MRI using higher in-plane resolution but this also resulted in a significant reduction in the signal-to-noise ratio of the T2-weighted MR images. Diffusion MRI revealed that water diffusion is highly anisotropic in the vascular cylinder. QSI and d-PGSE MRI showed that in the vascular cylinder some of the cells have sizes in the range of 6–10 μm. The MR images captured cell reorganization during adventitious root formation in the periphery of the primary vascular bundles, adjacent to the xylem pole that broke through the cortex and epidermis layers. This study demonstrates that MRI and diffusion MRI methods allow the non-invasive study of microstructural features of plants, and enable microstructural changes associated with adventitious root formation to be followed.

## Highlight

Magnetic resonance imaging (MRI) and diffusion MRI methods allow the non-invasive study of microstructural features of plants, and enable microstructural changes associated with adventitious root formation to be followed.

## Introduction

Plant tissues have complex microstructures that are tailored to their function. For example, the branched architecture of a plant’s root system is fundamental to its function in supporting plant productivity through both anchorage and uptake of nutrients and water (Van Norman, 2015). The adult plant root system consists of several types of roots, formed in different developmental contexts. Lateral roots branch from a primary seminal root, formed from the pericycle (Atta *et al.*, 2009), whereas adventitious roots develop on stems or leaves (Bellini *et al.*, 2014). In *Arabidopsis*, adventitious roots in the hypocotyl initiate from the pericycle cells adjacent to the xylem poles (Boerjan *et al.*, 1995).

Adventitious root formation is a complex process affected by multiple endogenous factors, including hormones and environmental factors (Blakesley, 1994). The plant hormone auxin plays a central role in adventitious root formation (Bellini *et al.*, 2014) as the combined activity of auxin influx and efflux carrier proteins generates auxin maxima and local gradients that inform root patterning (Xu *et al.*, 2005; Petrášek and Friml, 2009). Therefore, auxin is often applied exogenously to promote the development of adventitious roots on stem cuttings (Fogaça and Fett-Neto, 2005).

Lateral and adventitious root formation are well characterized in *Arabidopsis*, but there is limited knowledge on root formation in other species such as tomato. One of the reasons for this is that high-quality data can be obtained from florescence microscopy imaging only in relatively thin roots and hypocotyl tissues, such as those of *Arabidopsis*. However, light microscopy, with its relatively low penetration, is of limited value in the study of the thick roots and hypocotyl tissues of most crops (König, 2000; Benediktyová and Nedbal, 2009; Buda *et al.*, 2009). Since live imaging of inner developmental processes of crop plants via light microscopy remains challenging, researchers mostly depend on histology to observe initiation and growth processes that lack temporal resolution. Therefore, we sought to use magnetic resonance imaging (MRI), which has unlimited penetration of plant tissues, to study plant microstructure and microstructural changes associated with root growth and development.

MRI is a powerful technique for studying and gleaning macroscopic and microscopic information in a myriad of chemical and biological systems (Haacke *et al.*, 1999; Stark and Bradley, 1999). MRI was initially derived from nuclear magnetic resonance (NMR) and is a non-invasive technique capable of acquiring data from the entire sample in a non-destructive manner. This tremendous advantage of MRI has opened up a variety of applications in chemical, biomedical, and clinical sciences (Haacke *et al.*, 1999; Stark and Bradley, 1999; Blümich, 2003; Jones, 2010) and in plant research (for reviews see Köckenberger *et al.*, 2004; Van As *et al.*, 2009; Borisjuk *et al.*, 2012; Van As and van Duynhoven, 2013). However, MRI in plant sciences is still far from being a routine tool as it requires, in some cases, dedicated MRI methods and hardware (Van As and van Duynhoven, 2013; Metzner *et al.*, 2015; Nagata *et al.*, 2016). Despite the challenges, in recent years MRI has been used to image the growth of plants in their natural environment (Van As *et al.*, 2009; Borisjuk *et al.*, 2012; Van As and van Duynhoven, 2013), seed and bulb germination and development (Borisjuk *et al.*, 2012), water dynamics of the plant vascular system, and other processes (Van As, 2007; Windt *et al.*, 2009; Sibgatullin *et al.*, 2010; Dean *et al.*, 2014; Gruwel, 2014; Windt and Blümler, 2015; Nagata *et al.*, 2016; van Dusschoten *et al.*, 2016).

**Fig. 1.**
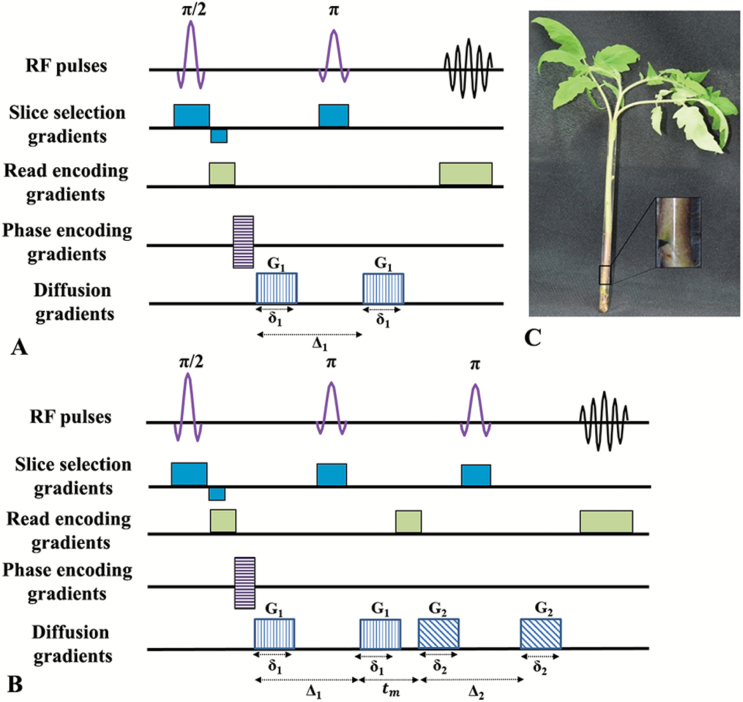
Diffusion MRI pulse sequences. (A) Single PGSE sequence, and (B) a double PGSE sequence. Note that by adding the diffusion sensitizing gradient-pulses (*G*) the relaxation-based sequence is transformed into the diffusion-based sequence. (C) Image of a horizontally cut whole tomato plant inserted to the NMR tube. Inset in (C) shows a magnification of the nick half-way across the stem to the mid-vasculature. (This figure is available in colour at *JXB* online.)

One of the main advantages of MRI is that image contrast depends on the pulse sequence used to collect the measured signal. Indeed, the contrast in MR images can be based on the different type of relaxations and on diffusion, perfusion, susceptibility, magnetization-transfer, chemical exchange saturation transfer, and more (Haacke *et al.*, 1999; Stark and Bradley, 1999; Blümich, 2003; Jones, 2010). The two commonly used types of MR images are relaxation-based and diffusion-based MR images. In relaxation-based MRI, longitudinal relaxation time (T1), transverse relaxation time (T2), and proton density (PD) MR images can be obtained just by changing the repetition time (TR) and the time-to-echo (TE) parameters. By changing the pulse sequence from spin-echo to gradient-echo the MR image becomes more susceptible to T2* (Stark and Bradley, 1999). The relaxation-based MRI sequences can be transformed into diffusion-based MRI sequences by adding diffusion-sensitizing gradient pulses as shown in Fig. 1 (Stejskal and Tanner, 1965; Wesbey *et al.*, 1984; Stark and Bradley, 1999; Price, 2009; Jones, 2010).

Diffusion, known also as Brownian motion, results from the random motion of molecules due to internal kinetic energy. The factors that can affect diffusion in a medium are the temperature, the viscosity of the medium, and the hydrodynamic radius of the diffusing particles. For molecules diffusing in a bulk solution, diffusion is Gaussian and isotropic, and the root mean square displacement (*rmsd*) appears equal in all directions and is given by the Einstein equation (Einstein, 1905):

$$rmsd=\sqrt{nDt_{\text{d}}}$$ (1)

where *n* is the dimension of motion (2, 4, and 6 for one-, two-, and three-dimensional cases, respectively), *D* is the diffusion coefficient, and *t*_d_ is the diffusion time.

The pulsed-field-gradient spin-echo (PGSE) sequence and its imaging version (Fig. 1A) (Stejskal and Tanner, 1965; Wesbey *et al.*, 1984, Price, 2009) are the most commonly used sequences for accurate, non-invasive measurement of diffusion coefficients. In such MR experiments, the signal attenuation, *E*(*q*), for molecular species exhibiting free diffusion is given by Eq. 2 (Stejskal and Tanner, 1965; Price 1997):

$$E\left(q\right)=\frac{S(q)}{S(q=0)}=e^{-4\pi^{2}{\left|q\right|}^{2}t_{d}D}=e^{-{\left(\gamma \delta G\right)}^{2}t_{d}D}=e^{-bD}$$ (2)

where the wave vector *q* is defined as:

$$q=\;{\left(\text{2}\pi \right)}^{-\text{1}}\delta \gamma G$$ (3)

In Eqs 2 and 3, *γ* is the gyromagnetic ratio, and *δ* and *G* are the pulse-gradient duration and intensity, respectively. When using rectangular gradients, *t*_d_ is equal to (Δ–*δ*/3) where *Δ* is the time between the edges of the two diffusion pulse-gradients. The *b*-value is therefore defined as:

$$b=–\text{4}\pi^{\text{2}}|q{|}^{\text{2}}t_{\text{d}}$$ (4)

When barriers are introduced into a sample, the diffusing particles may, after a sufficiently long diffusion time, encounter the restricting barriers and consequently the particles will exhibit restricted diffusion. At short diffusion times (*Δ*<<*l*^2^/2*D* where *l* is the size of the compartment), the diffusing particles do not diffuse far enough to probe the barriers of the compartment. As a result, the measured diffusion coefficient will be similar to that obtained for the free-diffusing particles. As diffusion time increases and reaches the order of, or surpasses *l*^2^/2*D*, an increasing fraction of diffusing particles will feel the effects of the boundary. Hence, the *rmsd* will not scale linearly with the square-root of the diffusion time, resulting in an apparent diffusion coefficient (ADC) (Price, 1997, 2009). At sufficiently long diffusion times, the diffusion of the particles becomes fully restricted by the boundaries and thus the *rmsd* becomes independent of the diffusion time (Price, 1997, 2009; Cohen *et al.*, 2016).

When diffusion is restricted (Tanner and Stejskal, 1968) only in some directions, diffusion is said to be anisotropic (Moseley *et al.*, 1990; Basser, 1995; Price, 1997, 2009; Jones, 2010; Shemesh *et al.*, 2010; Cohen *et al.*, 2016). Therefore, the extracted ADC values will depend significantly on the direction of the measurement, which can be easily controlled in diffusion MR experiments. Diffusion tensor imaging (DTI) is a method in which the diffusion coefficient in Eq. 2 is replaced by a tensor that describes the mobility of molecules in each direction (Basser, 1995; Le Bihan, 1995; Basser and Jones, 2002; Jones, 2010). In order to fully describe a diffusion tensor diffusion experiments should be performed along at least six non-collinear directions (Pierpaoli and Basser, 1996). A *b*_0_ MR image is also required, implying that at least seven MR images are required for DTI. DTI provides several parameters that are a reflection of the sample microstructure in each voxel. One of these parameters is the fractional anisotropy (FA), which demonstrates the degree of anisotropy in each voxel and varies from 0 (isotropic diffusion) to 1 (fully anisotropic diffusion). DTI also provides the mean diffusivity (MD), which describes the mean motion of the inspected molecules. DTI experiments are used extensively to study central nervous system (CNS) structure and pathologies (Le Bihan, 1995; Johansen-Berg and Behrens, 2009; Jones, 2010; Cohen *et al.*, 2016). In many DTI studies, at least in the CNS, the data has been analysed by assuming Gaussian diffusion and using the well-known Stejskal–Tanner equation (Eq. 2). DTI that has been heavily used in neuroscience and in clinical neurology (Basser and Jones, 2002; Le Bihan, 1995; Johansen-Berg and Behrens, 2009; Jones, 2010; Cohen *et al.*, 2016) has also been used to some extent in plant sciences (Van As *et al.*, 2009; Sibgatullin *et al.*, 2010; Gruwel *et al.*, 2013, Dean *et al.*, 2014; Gruwel, 2014). For example, Dean *et al.* (2014) used diffusion MRI and DTI to characterize developmental changes in grape berry, while Gruwel (2014) described how this methodology can be used to study plant roots.

However, in many cases of diffusion measurement the signal decay may appear to be non-Gaussian or non-mono-exponential, implying that the signal decay cannot be analysed using the Stejskal–Tanner equation. In such cases, different approaches are needed to analyse the data. One such approach is the model-free *q*-space approach that was originally developed by Callaghan and others to study porous materials and cell sizes (Callaghan *et al.*, 1990, 1991; Cory and Garroway, 1990). We expanded this approach to imaging of neuronal tissues and organs, to what is now known as *q*-space diffusion MRI or QSI (Assaf et al., 2000, 2002; Cohen and Assaf, 2002; Nossin-Manor *et al.*, 2002; Biton *et al.*, 2005, 2007; Bar-Shir *et al.*, 2009; Anaby *et al.*, 2013*a*, 2013*b*). QSI involves acquiring a series of diffusion-weighted MR images along a certain direction with increasing *q*-values. To analyse the experimental data the signal decay, *E*(*q*), is Fourier-transformed to afford the displacement probability function ${\bar{P}}_{s}\left(R,\Delta \right)$ for each pixel. This analysis is based on the Fourier relationship between ${\bar{P}}_{s}\left(R,\Delta \right)$ and (*q*, Δ):

$$E\left(q,\Delta \right)=\int^{\text{​}}{\bar{P}}_{s}\left(R,\Delta \right)\exp\left(i2\pi qR\right)dR$$ (5)

where *E*(*q*, Δ) represents the signal decay as a function of *q*, *R* is the net displacement vector and ${\bar{P}}_{s}\left(R,\Delta \right)$ is the displacement probability. The parameters obtained for each pixel in the image are the mean displacement, calculated from the full-width at half height (FWHH) of the displacement distribution profile, and the probability for zero displacement calculated from the height of the displacement profile (Assaf *et al.*, 2000; Cohen and Assaf, 2002). The major limitations of QSI are the requirement for relatively high diffusion weighting (strong gradients), resulting in relatively low signal-to-noise ratios (SNRs), and the need for a relative large number of *q*-values, resulting in long acquisition times. Note that QSI has been used to study compartment sizes in stems of asparagus (Lätt *et al.*, 2007).

The double-pulsed-field-gradient (d-PFG) MR methodology (Cory *et al.*, 1990; Mitra, 1995), recently referred to as a double-diffusion encoding (DDE) MR method (Shemesh *et al.*, 2016), is an additional diffusion MRI method for studying and obtaining information on tissue microstructure (Fig. 1B) and has been attracting more interest in recent years (Özarslan, 2009; Shemesh *et al.*, 2010, 2011, 2012*a*, 2012*b*; Finsterbusch, 2011; Özarslan *et al.*, 2011; Komlosh *et al.*, 2013). The d-PFG MR method is an extension of the well-known Stejskal–Tanner method and employs two diffusion gradient pairs, *G*_1_ and *G*_2_, with durations *δ*_1_ and *δ*_2_, respectively. The d-PFG MR sequence contains two diffusion time intervals, Δ_1_ and Δ_2_, between each gradient pair that may be separated by a mixing time, *t*_m_. In addition, these two gradient pairs may be applied collinearly (Cory *et al.*, 1990) or with an angle between them (Mitra, 1995), resulting in radial or angular d-PFG MR experiments, respectively.

When an angular d-PFG MR experiment is performed with a zero mixing time and a sufficiently long diffusion time to sense the restriction, the signal will exhibit angular dependency because of the microscopic anisotropy arising from the borders of the confined compartment (Mitra, 1995; Özarslan and Basser, 2008; Özarslan, 2009; Özarslan *et al.*, 2009). Therefore, zero mixing time angular d-PFG MR experiments have been suggested as an efficient tool for microstructural characterization in systems lacking macroscopic ordering or macroscopic anisotropy (Özarslan and Basser, 2008; Weber *et al.*, 2009; Shemesh *et al.*, 2010; Finsterbusch, 2011; Lawrenz and Finsterbusch, 2013). Double PFG MRS experiments have been used to study cell sizes in yeast cells and in celery (Özarslan *et al.*, 2011; Shemesh *et al.*, 2012*a*).

The purpose of the present study was to use relaxation-based MRI and, more importantly, diffusion-based MRI techniques to characterize tomato hypocotyl microstructure and to follow the early stages of the adventitious root initiation process non-invasively.

## Materials and methods

### Plant material

Plants of *Solanum lycopersicum* cv M82, sp^−^ were grown in soil in a climate-controlled chamber under long-day conditions (16 h light/8 h dark) at 25 °C.

### Imaging tomato hypocotyls

Five-week-old plants were imaged. Cotyledons and the first four leaves were removed to allow the insertion of the plant with its soil into the NMR tube. The meristem and young developing leaves were not damaged. Plants were inserted into NMR tubes filled with water (Fig. 1C). The tubes were then inserted into the MRI scanner such that the main axis of the plant was aligned along the main magnetic field (*z*-axis).

### Imaging adventitious root initiation

Eight-week-old or 5-week-old plants were cut horizontally half-way across the stem to the mid-vasculature, forming a nick. Cotyledons and the first four leaves were removed to allow the insertion of the plant into the NMR tube. The meristem and young developing leaves were not damaged. The tube was then filled with water and leaves were sprayed with solution containing 10 µM auxin (IAA) (Duchefa Biochmie 009552) and 0.05% Silwet L-77. Plants were scanned immediately. Between scanning sessions, plants were kept in the growth chamber.

### MRI

All MRI experiments were conducted on a 14.1 T wide-bore NMR scanner (Bruker, Karlsruhe, Germany) equipped with a Great 1 micro-imaging gradient system capable of producing pulsed-field gradients of up to 3 T m^–1^ in each direction.

### Experimental parameters for MRI of hypocotyl structure

All MRI experiments examining the hypocotyl structure were conducted with a slice thickness of 1 mm and a field of view (FOV) of 0.45 × 0.45 cm. Three continuous 1-mm slices were acquired with a protocol that included acquisition of T2 maps acquired with a multiple spin-echo Carr–Purcell–Meiboom–Gill-type MR imaging sequence, T1-weighted images, DTI, high *b*-value QSI and d-PGSE MR images. The T2 maps were collected with a TR of 3000 ms. The TE was varied between 10–160 ms, with 16 equal steps. The number of averages (NA) was 60. Two T2-weighted images (TE=10 ms and TE=50 ms) were extracted from the T2 map data. The T1-weighted images were collected with TR/TE of 700/15 and NA of 80. The relaxation-based MR images were collected with matrices of 128 × 64 or 256 × 128 (reconstructed to 128 × 128 or 256 × 256), resulting in in-plane spatial resolution of 35 × 70 µm^2^ or 17 × 35 µm^2^, respectively.

All diffusion-based MR images were collected with an in-plane resolution of 35 × 70 µm^2^. High *b*-value *q*-space diffusion MRI (QSI) images (Assaf *et al.*, 2000) were collected using a diffusion-stimulated echo sequence (STE) (Tanner, 1970) acquired in two different directions, perpendicular (*x*) and parallel (*z*) to the plant stem with diffusion time separation (Δ) of 15, 30, and 100 ms and a diffusion gradient duration (*δ*) of 2 ms. The diffusion gradient strength (*G*) was incremented in 16 equal steps for each direction from 2 to 600 mT m^–1^, resulting in a maximal *q*-value of 510 cm^−1^ and *b*-values of 1263 s mm^–2^, 2607 s mm^–2^, and 8981 s mm^–2^ for Δs of 15, 30, and 100 ms, respectively. The QSI data were acquired with TR/TE of 3000/23 and NA of 52, resulting in acquisition time of about 3 h per QSI image.

Diffusion tensor imaging (DTI) experiments were performed using a diffusion spin-echo sequence (SE) with the following parameters: TR/TE=3000/25, Δ/*δ*=15/2 ms. Thirty non-collinear gradient directions were collected with a *b*-value of 1263 s mm^–2^, and two additional images were collected with no diffusion weighting (*b*_0_). The number of echo planar imaging (EPI) segments (NS) and the NA were set to 4 and 28, respectively, resulting in total acquisition time of about 3 h per DTI data set.

The angular d-PFG MRI sequence used in the present study was based on a d-PGSE sequence (Mitra, 1995) with a finite mixing time (*t*_m_) that was integrated into a conventional EPI acquisition scheme (Shemesh *et al.*, 2010, 2011, 2012*b*). The angular d-PGSE MRI experiments were performed when the *G*_1_ gradient pair was fixed in the *x*-direction (perpendicular to main axis of the plant) and the orientation of *G*_2_, i.e. the second gradient pair, was varied in the *x*–*y* plane. The measurements were conducted with 13 different values of *φ* between 0° and 360°. The d-PGSE MRI data were collected with the following parameters: TR/TE=3000/40, *δ*_1_=*δ*_2_=2 ms, Δ_1_=Δ_2_=15 ms, *t*_m_=0 ms and |*G*_1_|=|*G*_2_|=525 mT m^−1^. These parameters resulted in a 2*q*-value of 894 cm^−1^ and a *b*-value of 967 s mm^–2^. The NS and NA values were 4 and 96, respectively, resulting in an acquisition time of about 4 h per d-PGSE MR data set.

### Experimental parameters for MRI of adventitious root

Additional MRI experiments were conducted with a slice thickness of 1 mm, a FOV of 1 × 1 cm or 0.8 × 0.8 cm, and in-plane resolution of 68 × 68 µm^2^ or 61 × 61 µm^2^, respectively. The MRI protocols for the T2 map images and T1-weighted images were similar to those used for analysis of hypocotyl structure, and only the number of averages was changed. The NA for the T2 maps and T1-weighted MR images were set to 16 and 40, respectively.

DTI experiments were performed with the following parameters: TR/TE of 3000/28 or 3000/23 with in-plane resolutions of 68 µm^2^ or 61 µm^2^, respectively, and Δ/*δ*=15/2 ms. Again, 30 non-collinear gradient directions were collected with a *b*-value of 967 s mm^–2^ together with two additional *b*_0_ images. The number of EPI segments (NS) and the NA were set to 4 and 10, respectively.

The angular d-PGSE MRI experiments were performed with the following parameters: TR/TE of 3000/41.5 or 3000/40 for in-plane resolution of 68 µm^2^ or 61 µm^2^, respectively, *δ*_1_=*δ*_2_=2 ms, Δ_1_=Δ_2_=15 ms, *t*_m_=0 ms and |*G*_1_|=|*G*_2_|=525 mT m^−1^. This resulted in a 2*q*-value of 894 cm^−1^ and a *b*-value of 967 s mm^–2^. The NS and NA values were 4 and 26, respectively. The total acquisition time was about 5 h for the entire MRI protocol.

To detect the formation of new roots the plants were scanned at four different time points; immediately and 2, 4, and 7 d after stem cutting and the hormone spray. Since MRI was conducted while plants were unacclimatized to a dark environment, we reduced the NA so that the MRI protocol would be as short as possible. The entire MRI protocol (T1, T2, DTI, and d-PFG MR imaging) lasted about 3.5 h. After undergoing MRI, the plants were transferred to a climate-controlled chamber.

### Data analysis

To obtain apparent T2 values, the raw data from the T2 mapping experiment were reconstructed to a symmetrical in-plane matrix and analysed using an in-house Matlab program tool. The DTI experiments were analysed using an ExploreDTI tool in Matlab^®^ to produce the FA and the MD maps. The QSI raw data were analysed according to the *q*-space approach described previously (Assaf *et al.*, 2000; Cohen and Assaf, 2002). First, the Fourier transformation was performed for each pixel in the image, resulting in a displacement distribution profile. Next, the displacement value, calculated from the FWHH of the displacement distribution profile, was extracted for each pixel and used to create displacement maps. The d-PGSE MRI data analysis and simulations were based on a method presented previously (Özarslan and Basser, 2008; Özarslan, 2009; Özarslan *et al.*, 2009; Shemesh *et al.*, 2010; Morozov *et al.*, 2015).

### Histological sections and microscopy

The MRI-scanned hypocotyls were fixed in FAA solution (50% ethanol, 5% acetic acid, and 3.7% formaldehyde) for at least 24 h, and then dehydrated using the tertiary butyl alcohol method (Johansen, 1940). The plant material was embedded in paraffin and cut into 8–12-μm thick cross-sections using a Microtome 820 (Spencer) (Johansen, 1940). Following paraffin removal by K-clear (KALTEK - Padova, Italy), the sections were mounted in glycerol and examined by light microscopy.

## Results

### Structural studies of the tomato hypocotyl

#### Conventional relaxation-based MRI

**Fig. 2.**
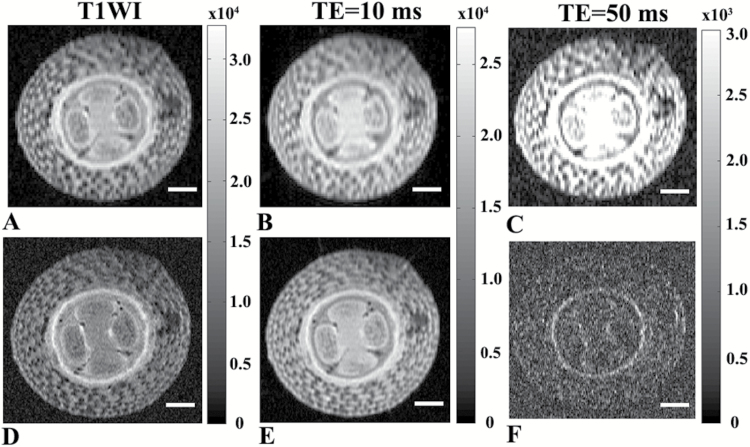
Relaxation-based MR images of tomato hypocotyls. Conventional MR images of 5-week-old tomato hypocotyls obtained with in-plane spatial resolutions of (A–C) 35 × 70 µm^2^ and (D-F) 17 × 35 µm^2^. (A, D) T1-weighed images collected with TR/TE of 700/15. (B, E) Short TE T2 MR images (TR/TE=3000/10), (C, F) Long TE T2-weighted images collected with TR/TE of 3000/50. Scale bars are 500 μm.

We first used conventional MRI to characterize the morphology of the tomato hypocotyl and to find the optimal in-plane resolution for the diffusion studies that provide the highest spatial resolution along with an adequate SNR at 14.1 T. Figure 2A–C shows the data obtained with an in-plane spatial resolution of 35 × 70 µm^2^ while Fig. 2D–F shows the same data at an in-plane resolution of 17 × 35 µm^2^. Slightly better contrast was observed in Fig. 2D–F as compared to Fig. 2A–C, but the SNRs of the higher-resolution images in Fig. 2D–F were significantly lower than that of the MR images in Fig. 2A–C. These images clearly demonstrate that the higher-resolution MR images have much lower SNRs, especially when the TE was set to 50 ms. Note also that almost no contrast was observed in the T2 maps that were computed (data not shown). In these images it is easy to distinguish between the pith and vascular cylinder areas as they differ in their contrasts. The epidermis and cortex layers with their large compartments are also clearly apparent. Signal intensity in relaxation-based MR images is a reflection of many physical parameters, such as proton density, T1, T2, susceptibility and other factors (Edzes *et al.*, 1998; Van As *et al.*, 2009). At high-spatial-resolution diffusion, susceptibility and other processes may affect the SNR and the contrast observed in long-TE relaxation-based MR images (Edzes *et al.*, 1998; Van As *et al.*, 2009). These MR images, however, do not provide detailed insights into the microstructural characteristics of the plant tissues. Therefore, we decided to revert to diffusion MR imaging, which is known to be more intimately associated with microstructural characteristics of the tissues.

### Diffusion-based MRI of the tomato hypocotyl

#### DTI studies

**Fig. 3.**
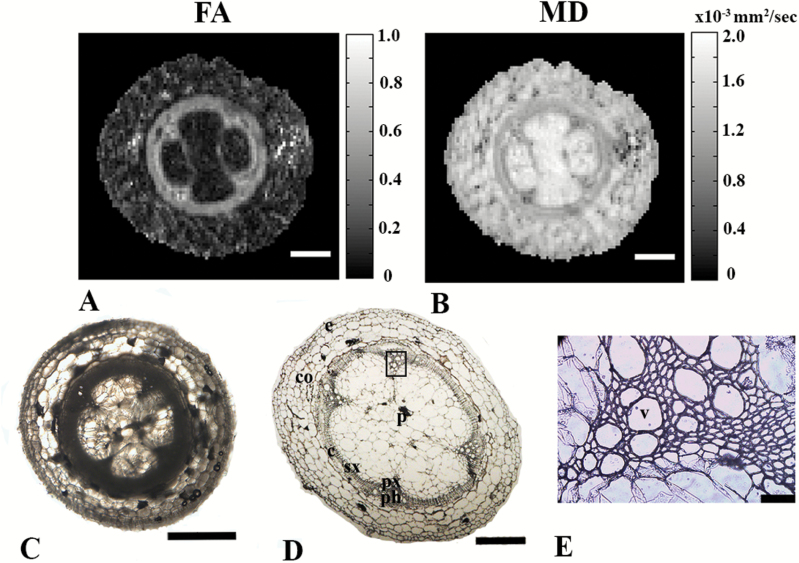
DTI-based MR images of tomato hypocotyls, including correlation with histology. (A) FA and (B) MD maps from DTI (*b*=1263 s mm^–2^, Δ=15 ms; scale bars are 500 μm). (C) Hand-cut- and (D) 12-μm histological cross-sections of 5-week-old tomato hypocotyls (bar are 1 mm and 500 μm, respectively), and (E) enlargement of the vascular bundle depicted on (D) (scale bar is 50 μm). The abbreviations used for the different regions in (D) and (E) are: px, primary xylem; sx, secondary xylem; ph, phloem; p, pith; c, cambium; co, cortex; e, epidermis; v, vessel.

To examine water diffusion inside the hypocotyl tissues and to get better insights into the structural characteristics of these tissues, we next conducted DTI experiments (Fig. 3). High FA values were observed in the vascular cylinder region, while low FA values in the range of about 0.2, reminiscent of that observed in grey matter of the CNS (Jones, 2010; Cohen *et al.*, 2016), were observed in other tissues of the tomato hypocotyl (Fig. 3A). This indicates that water diffusion in the phloem and xylem is indeed highly anisotropic. Moreover, low MD values were found in the vascular cylinder, while significantly higher MD values were observed in pith region (Fig. 3B). The FA and MD results shown in Fig. 3A and 3B are consistent with the histological maps obtained from a similar slice of the plant (Fig. 3C, D).

The histological images presented in Fig. 3C and 3D showed that most of the cells in the tomato pith and cortex were around 100 μm in diameter or larger. The cells in the epidermis were twice as small in size as those of the pith and cortex. The only areas where small cells of less than 10 μm were observed were in the vascular region, as shown in Fig. 3E which provides a magnification of the region highlighted in Fig. 3D. To obtain more direct insights into the physical sizes of cells in these areas of the sample it is possible, in principle, to use QSI. QSI generally uses higher diffusion weighting than DTI and has already been used to study neuronal tissue and other biological cells (Kuchel *et al.*, 1997; Assaf et al., 2000, 2002; Cohen and Assaf, 2002; Lätt *et al.*, 2007; Anaby *et al.*, 2013*a*, 2013*b*).

#### QSI studies

**Fig. 4.**
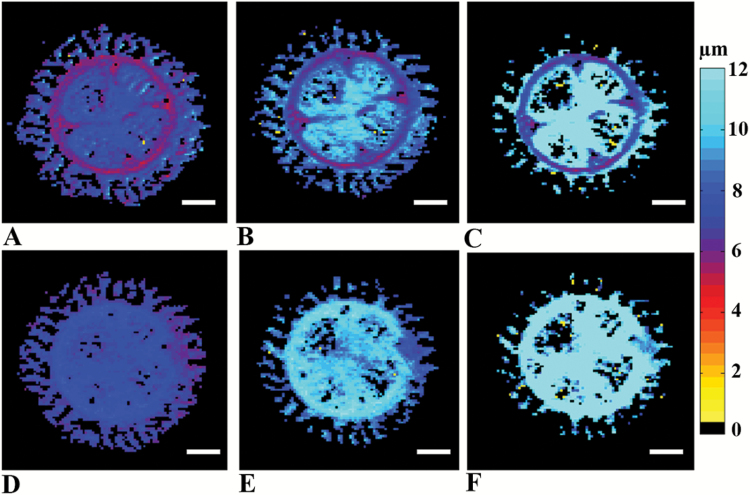
QSI-based images of tomato hypocotyls. Displacement maps of 5-week-old tomato hypocotyls extracted from high *b*-value QSI experiments performed in (A–C) the *x*-direction and (D–F) the *z*-direction, with three different diffusion times of (A, D) 15 ms, (B, E) 30 ms, and (C, F) 100 ms. Scale bars are 500 μm. (This figure is available in colour at *JXB* online.)

QSI studies were performed on the same 5-week-old tomato hypocotyls using a range of diffusion times in two orientations. Figure 4A–C shows displacement maps extracted from QSI studies performed in the *x*-direction, perpendicular to the main axis of the plant. These images show that water displacement increased with diffusion time in the epidermis, cortex, and pith cell types. However, the displacement values obtained from the vasculature region varied between 6 and 8 µm when the diffusion time was increased by more than a factor of 6. Figure 4D–F shows QSI displacement maps obtained when the diffusion was measured in the *z*-direction, parallel to the main axis of the plant. In this direction, the displacement values in the pith, cortex, and epidermis appeared similar to those obtained from QSI experiments performed in the *x*-direction (compare Fig. 4D–F with 4A–C). Interestingly, in the QSI studies performed in the *z*-direction no further water restriction was observed in the vascular cylinder region and the displacement maps appeared nearly isotropic across the entire cross-section of the hypocotyl. Thus, in the *z*-direction diffusion appears nearly homogenous across the tomato hypocotyl.

#### d-PGSE MRI studies

**Fig. 5.**
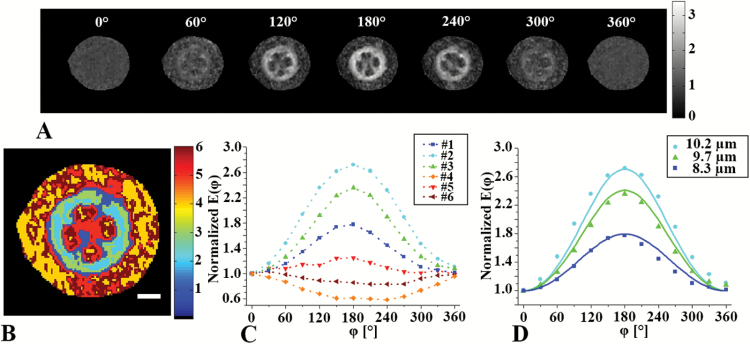
d-PGSE MRI analyses of tomato hypocotyls. (A) Raw data from the angular d-PGSE MRI experiment with zero mixing time conducted on 5-week-old tomato hypocotyls when *φ* was rotated in the *x*–*y* plane (i.e. perpendicular to the main axis of the plant). (B) *k*-means clustering analysis of the data presented in (A); The scale bar is 500 μm. (C) Normalized *E*(*φ*) profiles of the clusters shown in (B). (D) Normalized *E*(*φ*) profiles obtained from clusters in the vascular bundle region (symbols). Simulated curves are shows as lines for the indicated sizes. (This figure is available in colour at *JXB* online.)

We next performed angular d-PGSE MRI studies on 5-week-old tomato hypocotyls with zero mixing time in the *x*–*y* plane (i.e. perpendicular to the main axis of the plant). Figure 5A shows the angle dependency of the normalized signal, *E*(* φ *), of the hypocotyl. Only the vascular cylinder cells showed a bell-shaped signal dependency, and the signal in these areas was found to increase to from 0° to 180° followed by a decrease to 360° (Fig. 5A). Interestingly, no angular dependencies were observed in other hypocotyl regions. Figure 5B and 5C present signal profiles and a *k*-means clustering segmentation of the different tissues of the hypocotyl into six different clusters. The six clusters identified were found to have very different angle dependencies of *E*(* φ *)_._ Clusters 4–6 showed nearly no angle dependencies of *E*(* φ *)_,_ reminiscent of free diffusion. However, clusters 1–3 showed the expected bell-shaped signal dependency (Fig. 5C). Figure 5D shows the normalized *E*(* φ *) profiles along with the respective simulations for the three clusters originating from the vascular cylinder region (xylem and phloem areas). Very good agreement was found between the simulations and the experimental data. Based on these analyses, cell diameters in the range of 8 to 10 µm were extracted (Fig. 5D). These sizes are in good agreement with the cell diameters in this area as seen using histology (Fig. 3E).

### MRI of adventitious root formation

To promote adventive root formation, we generated artificial auxin maxima at the scanning position. Eight-week-old tomato plants were treated with a solution containing 10 µM auxin (sprayed on developing leaf primordia) and the hypocotyls were cut horizontally half-way across the stem (1.5 mm), generating a nick up to the mid-vasculature and resulting in accumulation of auxin above the site of the cut. The tomato plants were immersed whole into water to allow the development of adventitious roots.

**Fig. 6.**
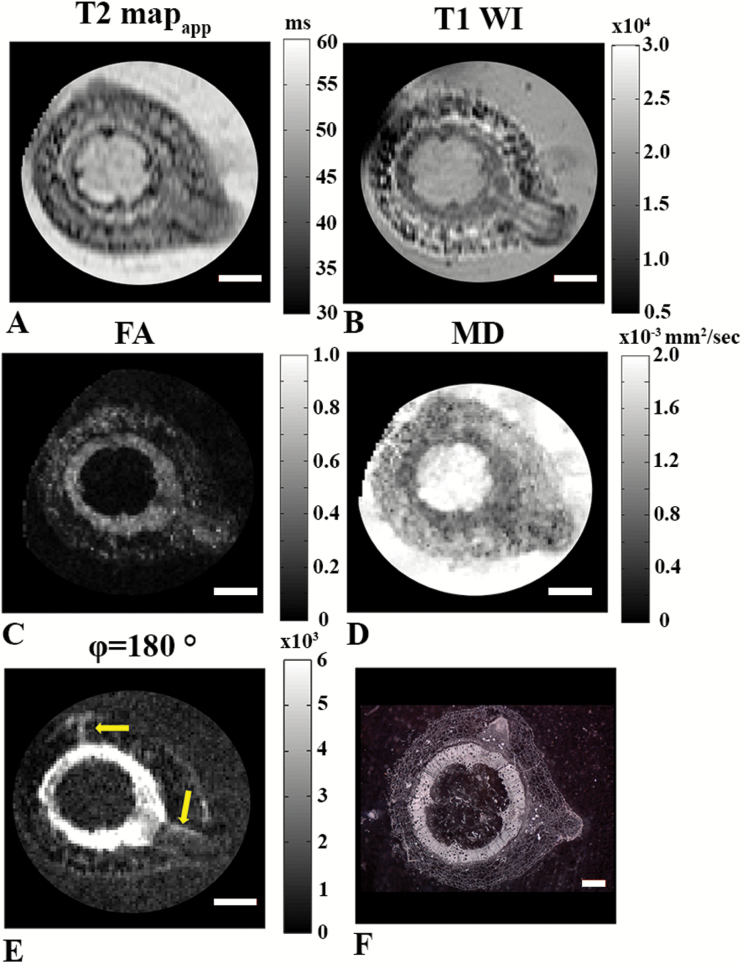
MR images obtained 24 h after adventitious root formation in 8-week-old plants (in-plane resolution of 68 µm^2^). (A) Apparent T2 map (TR/TE=3000/10–160). (B) T1-weighted image (TR/TE=700/15). (C) FA image. (D) MD image from DTI (*b*=1263 s mm^–2^, Δ=15 ms). (E) d-PGSE MR image for *φ* of 180°. Regions of small cells are indicated by the arrows. Scale bars in (A–E) are 1 mm. (F) Histological section at a slightly different location of the same hypocotyl; the scale bar is 500 µm.

The MR images presented in Fig. 6 were collected 24 h after generation of the artificial auxin maxima. These images show that the new roots initiate from the vascular bundle periphery/pericycle region and break their way out through the cortex and epidermis cells. This is visible on the apparent T2-map and the T1-weighted MR image (Fig. 6A and B). However, more significant contrast between the developing adventitious roots and the cortex tissues is apparent on the T1-weighted MR image. Note that in this case the in-plane resolution was significantly lower than in Figs 2–5, resulting in higher SNR. Figure 6C and 6D show that, at this developmental stage, the FA and MD values obtained for adventitious roots were more similar to those of the vascular bundles (xylem and phloem areas) than to cortex and epidermis. Moreover, the bundle region around the initiation site of the adventitious root seems to be wider than in other areas of the vascular bundle and secondary xylem perimeter. Figure 6E also shows the zero-mixing time d-PGSE MR image for *φ* of 180° collected on the same plant. In this MR image, regions consisting of relatively small cells were observed mostly in the vascular cylinder, namely the primary bundles, cambium, phloem, and secondary xylem (see also Fig. 5), and seemingly in some areas of the adventitious roots (Fig. 6E, arrows). Figure 6F presents the histological section obtained from the same tomato hypocotyl at a similar development stage but at slightly different plane. In this stage of development, some of the cells of the initiating adventitious root were smaller than other cells in the cortex but appeared larger compared to the vascular cylinder cells. This may be the reason why the adventitious root cells appeared less intense in the FA map obtained from DTI (Fig. 6C) and in the zero-mixing time d-PGSE MR image (Fig. 6E).

**Fig. 7.**
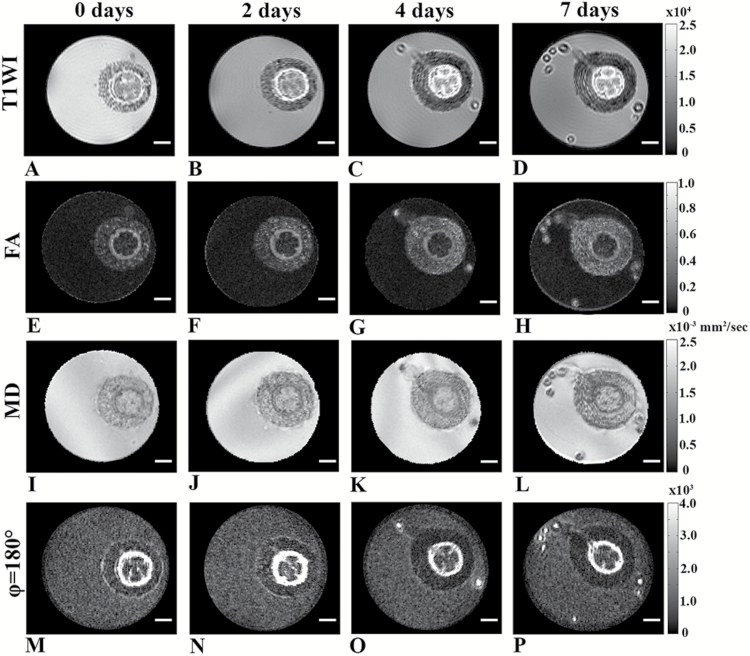
Time-course of adventitious root formation in 5-week-old plants by live MRI imaging (in-plane resolution of 61 µm^2^). (A–D) T1-weighted images, (E–H) FA images, and (I–L) MD maps obtained from DTI. (M–P) Zero mixing time d-PGSE MR images for *φ* of 180° map. All scale bars are 1 mm.

Next, we followed the time-course of the formation of adventitious root by MR imaging. The base of hypocotyls of 5-week-old tomato plants were cut to form a nick to the mid-vasculature. Plants were then inserted into NMR tubes filled with water. The MRI protocol was shorter than that used to collect the data shown in Fig. 6 in order to minimize the effect of the MRI measurement on development, resulting in somewhat lower SNRs in the different MR images collected. During the first two days after nicking, no significant tissue changes were observed under these imaging conditions (Fig. 7). The formation of new adventitious roots, however, was apparent at day 4 after induction of adventive root formation. Root formation was observed in all the MR images collected at this time point. These images and those collected at day 7 showed that the adventitious root that were formed initiated from the primary vascular bundle periphery region (peri-vasculature parenchyma cells/pericycle) and then developed through the cortex. Interestingly, cell proliferation was observed at days 4 and 7; however, some tissue reorganization was observed even at day 2. These MR data indicate that some structural changes occurred in the vasculature areas at the early stages of adventitious root formation.

## Discussion

In this work, we have shown that MRI, and more specifically advanced diffusion MRI, is able to characterize some facets of hypocotyl microstructure and can provide significant insights into the process of adventitious root formation in a non-invasive manner.

First, we conducted relaxation-based MRI by collecting T1-weighted and T2-weighted MR images at two different in-plane resolutions (Fig. 2). The MR images collected with higher in-plane resolution showed, as expected, slightly more detailed structural information for the hypocotyl tissue, although with low SNRs. The cortex cells were easily visualized at both in-plane resolutions, and were observed to be polygonal and closely packed. The sizes of the vascular bundle cells could not be visualized directly by the T1-weighted and the T2-weighted MR images at the spatial resolutions used in this study, since many of these cells are significantly smaller than cortex cells, having diameters in the range of 8–12 µm (Fig. 4D). The cells of the pith region are similar in diameter or even larger than the cells in the cortex, but these cells were not clearly apparent on the T1-weighted and T2-weighted MR images, and consequently the pith region appears hyper-intense compared to the vascular bundle area.

Water molecules have relatively long T1 and T2 values, lipids in membranes have short T1 and T2 values, and macromolecules generally have long T1 and short T2 values. Therefore, it is not surprising that these relaxation properties are used as contrast in MRI. We found a high signal inside the cortex cells but, as expected, less signal between the cells since the membranes have relatively lower water content and may have short T2 values. Moreover, the T2 MR maps computed (data not showed) had low SNRs, especially those with the higher in-plane resolution. The images presented in Fig. 2C and 2F indicate that the contrast and even the SNR in the relaxation-based MR images at high resolution and long TE are to some extent also affected by susceptibility, diffusion, and other effects related to the MR acquisition scheme, such as the high read-out gradients used (Edzes *et al.*, 1998; Van As *et al.*, 2009). Therefore, the apparent T2 values extracted from such MR experiments are usually smaller than the intrinsic ones. These effects become even larger at high magnetic fields and at high in-plane resolution of the kind used in the present study. The low SNRs observed in the long TE T2-weighted MR images with the higher resolution led us to prefer the use of the lower in-plane resolution in the diffusion MR images we collected next.

To obtain microstructural information on the hypocotyl tissues we collected advanced diffusion MRI data. First, we collected DTI data as shown in Fig. 3. High FA and low MD values were extracted from DTI in the vascular cylinder region as compared to other regions of the hypocotyl. These results imply that water diffusion in these bundles is anisotropic and can be probed with moderate diffusion time. This anisotropy probably originates from the highly packed, coherently organized, elongated cells in these areas. Interestingly, the MD values in pith region were found to be high and reminiscent of free water, indicating that water molecules inside the pith region exhibit mostly free diffusion under the experimental conditions used. The DTI results obtained were consistent with the histological image obtained from a similar slice of the plant, indicating that DTI indices could potentially be used as biomarkers for hypocotyl macroscopic organization. However, DTI, which is based on the assumption that water diffusion is Gaussian, is less suitable to describe diffusion in confined geometries and to provide specific, quantitative microstructural information. To obtain such information we performed QSI experiments with different diffusion times and in different directions of the hypocotyl (Fig. 4). When the QSI experiments, collected with a stimulated echo-based diffusion sequence, were conducted along the *x*-direction (i.e. perpendicular to the main axis of the hypocotyl) an increase in the diffusion time resulted in larger water displacement both in cortex and pith areas. In the vascular bundle regions, however, only a very small increase in the mean displacement was observed with the increase in diffusion time. The displacement values obtained from the vascular regions remained almost without change and varied between 6–8 µm when the diffusion time was varied from 15 to 100 ms. This indicates that in the vascular bundle regions, water diffusion in the direction perpendicular to the main hypocotyl axis is quite restricted. The fact that some increase in the mean displacement and in the extracted sizes was observed when the diffusion time was increased suggests that the membranes are not completely impermeable (Sibgatullin *et al.*, 2010). Note that to probe water restriction in cortex and pith cells, the size of which are 100 µm or more, extremely long diffusion times in the region of 2.5 s should be used. These diffusion times are impractical and are much longer than those applied in the present study since we have used a 14.1-T instrument, where susceptibility effects are significant and apparent T2s are relatively short. At a lower magnetic field (3 T), however, it is possible to perform diffusion measurements with diffusion times of up to 1 s (Sibgatullin *et al.*, 2010). When QSI experiments were conducted parallel to the main axis of the plant, very similar displacement values were obtained for all regions including the vascular bundles at all diffusion times, resulting in relatively smooth displacement maps. Moreover, in this direction the displacement increased nearly linearly with the square-root of the diffusion time, suggesting that water diffusion is essentially free parallel to the main axis of the plant. These results indicate that the vascular bundle region consists mainly of coherently organized and highly packed cells with cylindrical geometry. In contrast, the QSI data seem to suggest that most cells of the cortex and pith are relatively large and probably spherical.

To further characterize the hypocotyl microstructure, angular d-PGSE MRI experiments with zero mixing times were conducted in the *x*–*y* plane, perpendicular to the main axis of the same plant (Fig. 5). The raw images obtained showed a bell-shaped signal dependency only for signals originating from the vascular bundles. The signal of the bundle cells increased from 0° to 180° and then decreased from 180° to 360°. Interestingly, no angular dependency was obtained for signals originating from other plant regions. This is in accordance with the results from DTI, QSI, and histology, which showed that the cells in the cortex and the pith tissues are large, making it impossible, with the experimental conditions used in the present study, to probe restriction of water diffusion there. The clustering analysis showed different angular dependencies in different cells in the vascular cylinder, suggesting that angular d-PGSE MRI has the ability to differentiate between different areas and, more importantly, to segment the vascular bundle into regions based on cell size. Simulations of the d-PFG MRI experimental signals were found to be in good agreement with the experimental data and cell sizes in the range of 8–10 µm were extracted. It is interesting to note that the cells in the vascular cylinder are organized into three circular clusters. On average, cells in the interior of the vascular bundles were the largest, and the smaller cells were observed in the outer periphery of the vascular cylinder. These observations are consistent with the histology results presented in Fig. 3D.

As MRI can provide structural information and advanced diffusion MRI can reveal microstructural features in plant tissues non-invasively, we sought to visualize adventitious root initiation using these MRI techniques (Fig. 6). This was an even more difficult task as we had to use plants with smaller hypocotyl cross-sections, and because the plants had to be immersed in water when we collected the MRI data. The latter resulted in a high background signal in some of the MR images. The MRI data collected following nicking of the hypocotyl to initiate adventive root formation showed that the new roots emerged from the primary vascular bundles and out through the cortex. Low FA and high MD values were found in the pith region of the plant and, interestingly, the FA and MD values of initiating adventitious roots were more similar to those of vascular bundle cells, indicating that the newly formed cells were relatively small or densely packed. Moreover, we observed that the vascular bundles thickened where the adventitious roots started to occur, suggestive of high levels of cellular proliferation in the region of root initiation. In addition, both the FA from DTI and d-PGSE MR (*φ*=180°) images seemed to indicate that some reorganization occurred in the vascular bundles where the adventitious roots developed (Fig. 6C and 6E). Our data indicate more isotropic diffusion in the area of root development, which may originate from the formation of more isotropic or larger cells. Histological images obtained from similar tissue were found to be in good agreement with the MRI findings. Histology and MRI suggested that some of the cells in the initiating adventitious root were somewhat smaller than those of cortex, but probably larger than the cells in the vascular cylinder.

After characterizing the structural changes associated with adventitious root formation, we followed the time-course of this process by live MRI imaging (Fig. 7). This was an even more challenging task since we had to shorten the MRI protocol, which resulted in lower SNRs in the obtained images. The formation of new adventitious roots was detected in all MR images after 4 d. The roots initiated from the vascular bundle regions, developed through the cortex, and broke out through the hypocotyl epidermis. Interestingly, cell proliferation that resulted in structural changes was not observed after 2 d (Fig. 7). The time-course of the development of the adventitious roots was slower inside than outside the magnet. This may be the result of the stress imposed on the plant in the magnetic field and, more importantly, due to the fact that our MRI measurements were performed in a high-resolution MRI scanner where the plants were in the dark. For plants that were not in the magnet, root development was found to be faster. These observations show that the high-field MRI scanner used in the present study, which provided a wealth of structural information and some microstructural features of the plant tissues, is less suitable for studying time-courses of plant physiology since the measurement itself seems to affect to some extent the time-course of the natural development of the roots. A partial solution would be to provide illumination within the magnet or to use a dedicated MRI system with a magnet that allows performing such study in the natural environment. Indeed, in recent years several such MRI systems, most of which have relatively low fields, have been described in the literature (Van As, 2007; Nagata *et al.*, 2016). However, these instruments generally have lower sensitivity and homogeneity compared with the MRI scanner used here, making it more difficult to implement advanced MRI techniques.

There are additional methods for plant visualization, such as micro-CT, cryo-sem (Choat *et al.*, 2015; Umebayashi *et al.*, 2016), or positron emission tomography (PET) (Converse *et al.*, 2015). However, MRI has several important advantages, the main one being that, compared to other imaging methods, MRI is a true multi-parametric method where the image contrast can provide information on a range of tissue characteristics, from proton density and relaxation, through to susceptibility, diffusion, and flow. In addition, MRI has an unlimited penetration to plant tissues, uses safe RF pulses, and is less invasive and more environmentally friendly. We therefore anticipate that MRI will contribute more to plant research in the future.

## Conclusions

In this study, we show that multi-parametric MRI methods and advanced diffusion MRI methods may provide important information on plant tissues. Diffusion MRI techniques provide powerful tools for characterizing, with great detail and non-invasively, structure and microstructural features of the plant tissues as well as developmental processes in live plants. We show that diffusion MRI techniques, such as DTI, QSI, and d-PGSE, provide unique tools to study the macro- and microscopic organization of the plant and, after proper modelling, allow the estimation of the sizes of some of the plant cells with high accuracy, thus providing a novel dynamic spatiotemporal ‘virtual histology’. The main advantages of MRI are its non-invasiveness and the fact that many different images can be acquired, each sensitive to different sets of physical parameters reflecting different physiological or biological process. Therefore, we believe that multi-parametric MRI and diffusion MRI have the potential for following complex biological processes in plants.
